# Global epidemiology of Familial Mediterranean fever mutations using population exome sequences

**DOI:** 10.1002/mgg3.140

**Published:** 2015-04-05

**Authors:** Kohei Fujikura

**Affiliations:** Kobe University School of Medicine7-5-1, Kusunoki-cho, Chuo-ku, Kobe, 650-0017, Japan

**Keywords:** Allele frequency, epidemiology, exome, Familial Mediterranean fever, genetic diagnosis

## Abstract

Familial Mediterranean fever (FMF) is an inherited disorder characterized by recurrent episodes of fever accompanied by sterile peritonitis, arthritis, and pleuritis. Many mutations in the *MEFV* gene have been identified as causing FMF. However, accompanying epidemiological information remains quite scarce except in some Mediterranean countries, and the degree of penetrance has been a subject of controversy. Here, I established a genetic epidemiology of full FMF mutations using two population exome studies. Of 57 mutations associated with FMF, 22 were detected in a total of 9007 individuals from two exome sequences. Exome-based epidemiology revealed the carrier rates of FMF in 28 populations in 19 countries by individual mutation and showed strong population specificity for the *MEFV* mutations. Unexpectedly high carrier rates suggested that some mutations are benign variants with no pathological significance and highlighted the need for caution in analyzing *MEFV* mutations. Similar approach could be used to uncover the incomplete or no penetrance of mutations in most inherited disorders.

## Introduction

Familial Mediterranean fever (FMF; MIM# 249100) is an inherited disorder characterized by recurrent short episodes of fever, sterile peritonitis, arthritis, and pleurisy (Sohar et al. 1967; Livneh et al. 1997; Ben-Chetrit and Levy 1998). The febrile attacks are accompanied by a strong acute phase response, and the most severe complication is the development of renal amyloidosis (Sohar et al. 1967; Livneh et al. 1997; Ben-Chetrit and Levy 1998). FMF occurs most commonly among people from the Mediterranean basin (such as non-Ashkenazi Jews (Lazarin et al. 2013), Arabs (Majeed et al. 2005), Armenians (Cazeneuve et al. 1999), Greece (Konstantopoulos et al. 2003), and Turks (Dundar et al. 2011; Neocleous et al. 2015), and also in other countries (Sohar et al. 1967; Ben-Chetrit and Levy 1998).

The causative gene for FMF, *MEFV*, was first identified by two independent groups in 1997 (The French International FMF consortium 1997; The International FMF Consortium 1997). The *MEFV* gene is located on chromosome 16p13.3 and encodes a 781-amino acid protein (10 exons) known as pyrin (The French International FMF consortium 1997; The International FMF Consortium 1997). Previous studies on FMF patients and animal models suggest that *MEFV* mutations lead to gain of pyrin function, resulting in increased IL-1*β* secretion by monocytes and a prolonged inflammatory response when stimulated with lipopolysaccharide (Chae et al. 2011; Omenetti et al. 2014).

To date, over 50 *MEFV* mutations have been identified in FMF patients (Touitou 2001; Giancane et al. 2015). The five founder mutations, E148Q, M680I, M694I, M694V, and V726A, were reported to account for approximate 70% of cases of FMF from the Mediterranean origin and the nonfounder mutations would constitute the remaining proportion (Touitou 2001) although the pathogenic role of some mutations including E148Q remains debatable (Tchernitchko et al. 2003, 2006; Giancane et al. 2015). Draft guidelines for the genetic diagnosis of FMF were also prepared based on the current practice and data from the literature (Shinar et al. 2012). However, despite the recent advances in FMF studies, the epidemiological information is still insufficient and inconclusive, except in some Mediterranean countries. In addition, the degree of penetrance of many mutations has not been a subject of research for many years. The aim in this study was to establish the global epidemiology of autosomal recessive FMF (MIM# 249100) mutations using the population exome sequences and to evaluate the penetrance of each *MEFV* mutation.

## Methods

### Analysis of genetic variants using two representative exome projects

Genetic pipelines from 1000G (www.ncbi.nlm.nih.gov/variation/tools/1000genomes/) and NHLBI (http://www.nhlbi.nih.gov/) datasets were collected in VCF format. The datasets consisted of a total of 18,014 alleles from high-coverage exome sequences derived from 28 ethnic groups in 19 countries. The *MEFV* mutations for FMF were retrieved and selected from literature sources in PubMed (www.ncbi.nlm.nih.gov/pubmed), OMIM (http://www.ncbi.nlm.nih.gov/omim) and the autoinflammatory mutation database INFEVERS (http://fmf.igh.cnrs.fr/ISSAID/infevers/index.php) (Milhavet et al. 2008). The detected variants were then classified by pathogenicity, mutation type, allele frequency, countries, and racial groups. Information on mutation types, positions, reference sequences, and pathogenicity was retrieved from OMIM and NCBI dbSNP (http://www.nlm.nih.gov/SNP/) to generate exome-based epidemiology. ExAC Browser (http://exac.broadinstitute.org/) was additionally searched for the mutation alleles of *MEFV*.

### Pairwise proportion tests of data consistency between two different exome resources

To project the performance of risk prediction based on analyses of exome sequence studies, exome-based estimates were statistically compared with the clinical prevalence survey. Evidence of data consistency was based on significant differences in pairwise comparisons between populations if two estimates differed significantly (two-sample test for equality of proportions with continuity correction). The standard hypothesis test was *H*_0_: *π*_1_ = *π*_2_ against the alternative (two-sided) 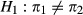. The pairwise prop test can be used to test the null hypothesis that the proportions (probabilities of success) in two groups are the same. This test is referred to as a *z*-test because the statistics were as follows:

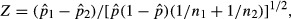
where 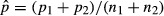 and indices (1, 2) refer to the first and second line of the table. In a two-way contingency table where *H*_0_: *π*_1_ = *π*_2_, this should yield comparable results to those of the ordinary *χ*^2^ test.

## Results

### Strategy for epidemiological research on *MEFV* mutations using population exome sequences

**Figure 1:**
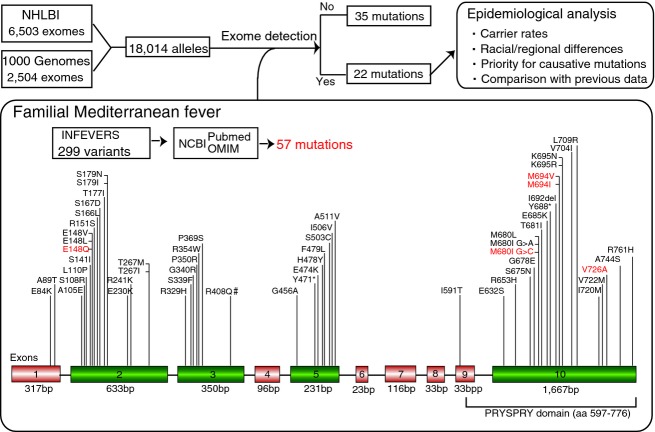
Strategy of epidemiological research on FMF using population exome sequences. A flowchart used to study epidemiology shows the process of mutation detection using the 1000G and NHLBI datasets. A total of 18,014 alleles were screened for 57 *MEFV* mutations linked to FMF. A schematic representation of FMF-associated mutations in the *MEFV* gene is shown below. *MEFV* mutations most frequently found in FMF patients reside in exon 10 (depicted in green), which encodes the C-terminal PRYSPRY domain. Exons 2, 3, and 5 contain a substantial number of rare mutations (depicted in green). The five founder mutations are indicated in red. # R408Q and P369S mutations had been reported in cis as a single allele resulting in a variable clinical symptoms and R408Q is not likely to be a disease-causing variant (Bell et al. 2011).

As a first step toward determining the genetic epidemiology of recessive FMF, genotyping datasets were collected from the 1000G and NHLBI projects for the causative variations (Fig.1). The datasets included the exome and its surrounding intronic sequences for 2504 individuals of 26 ethnic origins (ACB, African Caribbeans in Barbados; ASW, American’s of African Ancestry in SW; BEB, Bengali from Bangladesh; CEU, Utah Residents (CEPH) with Northern and Western European ancestry; CHB, Han Chinese in Beijing, China; CHS, Southern Han Chinese; CDX, Chinese Dai in Xishuangbanna, China; CLM, Colombian from Medellin; ESN, Esan in Nigeria ; FIN, Finnish in Finland; GBR, British in England; GIH, Gujarati Indian from Houston, Texas; GWD, Gambian in Western Divisions in The Gambia; IBS, Iberian population in Spain; ITU, Indian Telugu from the UK; JPT, Japanese in Tokyo, Japan; KHV, Kinh in Ho Chi Minh City, Vietnam; LWK, Luhya in Webuye; MSL, Mende in Sierra Leone; MXL, Mexican ancestry from Los Angeles; PEL, Peruvians from Lima, Peru; PJL, Punjabi from Lahore, Pakistan; PUR, Puerto Rico from Puerto Rica; STU, Sri Lankan Tamil from the UK; TSI, Toscani in Italia; YRI, Yoruba in Ibadan) and 6503 individuals of two ethnic origins (AA, African Americans; EA, European Americans) (Table1). Caucasians constituted 20.1% and 66.1% of subjects from the 1000G and NHLBI groups, respectively, whereas Africans constituted 26.4% and 33.9% of subjects, respectively (Table1). East Asian, South Asian, and Hispanic populations, which were represented only in the 1000G project, constituted 20.1%, 19.5%, and 13.9% of the group, respectively (Table1). Six samples were from within the United States, and others were from 16 countries, such as China, Japan, Colombia, Mexico, Puerto Rico, England, Germany, Kenya, and so on (Table1).

**Table 1:** Population disposition (ethnicity and male/female ratio)

Population	Total no.	Percentage (in each project)	Percentage (in total)
1000 genomes			
AFR (African)	661	26.4	7.34
AMR (Ad Mixed American)	347	13.9	3.85
EAS (East Asian)	504	20.1	5.60
EUR (European)	503	20.1	5.58
SAS (South Asian)	489	19.5	5.43
ACB (African Caribbeans in Barbados)	96	3.83	1.07
ASW (Americans of African Ancestry in SW USA)	61	2.44	0.68
BEB (Bengali from Bangladesh)	86	3.43	0.95
CDX (Chinese Dai in Xishuangbanna, China)	93	3.71	1.03
CEU (Utah Residents (CEPH) with Northern and Western European ancestry)	99	3.95	1.10
CHB (Han Chinese in Bejing, China)	103	4.11	1.14
CHS (Southern Han Chinese)	105	4.19	1.17
CLM (Colombians from Medellin, Colombia)	94	3.75	1.04
ESN (Esan in Nigeria)	99	3.95	1.10
FIN (Finnish in Finland)	99	3.95	1.10
GBR (British in England and Scotland)	91	3.63	1.01
GIH (Gujarati Indian from Houston, Texas)	103	4.11	1.14
GWD (Gambian in Western Divisions in The Gambia)	113	4.51	1.25
IBS (Iberian population in Spain)	107	4.27	1.19
ITU (Indian Telugu from the UK)	102	4.07	1.13
JPT (Japanese in Tokyo, Japan)	104	4.15	1.15
KHV (Kinh in Ho Chi Minh City, Vietnam)	99	3.95	1.10
LWK (Luhya in Webuye, Kenya)	99	3.95	1.10
MSL (Mende in Sierra Leone)	85	3.39	0.94
MXL (Mexican Ancestry from Los Angeles USA)	64	2.56	0.71
PEL (Peruvians from Lima, Peru)	85	3.39	0.94
PJL (Punjabi from Lahore, Pakistan)	96	3.83	1.07
PUR (Puerto Ricans from Puerto Rico)	104	4.15	1.15
STU (Sri Lankan Tamil from the UK)	102	4.07	1.13
TSI (Toscani in Italia)	107	4.27	1.19
YRI (Yoruba in Ibadan, Nigeria)	108	4.31	1.20
NHLBI			
EA (European American)	4300	66.1	47.7
AA (African American)	2203	33.9	24.5
Total	9007		100

Genetic screening for a possible carrier state was indicated for all individuals. A total of 299 (September 2014) nucleotide variants in *MEFV* have been reported in INFEVERS (Milhavet et al. 2008), but only a subset is found in patients with typical FMF disease and others are benign polymorphisms including synonymous and nonsynonymous variants. Of 57 *MEFV* mutations for FMF, 22 were detected in one or both of the two population exome datasets (Fig.1) (the list of *MEFV* mutations was retrieved from NCBI OMIM, INFEVERS, and PubMed; see also supplementary references). These mutations were present above the minimum variant allele frequency threshold (5.55E-05). *MEFV* mutations were classified by mutation type, derived allele frequency (DAF), countries, racial group, and clinical impact (Fig.1 and Table2). The other 35 mutations were not detected owing to their low DAF in a total of 9007 individuals (Fig.1).

**Table 2:** Estimated carrier rates of FMF by race, ethnicity, and country using 1000G and NHLBI

Disease name	Familial mediterranean fever (FMF)						
OMIM entry	#249100						
Gene name	MEFV						
mRNA ID	NM_024596.3						
Variant name	p.Glu84Lys	p.Leu110Pro	p.Glu148Gln	p.Arg151Ser	p.Glu230Lys	p.Thr267Ile	p.Arg329His
dbSNP	rs150819742	rs11466018	rs3743930	rs104895185	rs104895080	rs104895081	rs104895112
ALL	0.000167 (3/17996)	0.00330 (59/17878)	0.0440 (770/17494)	0.000225 (4/17778)	0.000392 (7/17870)	0.000111 (2/18002)	0.00178 (32/18002)
1 in _	5998.7	303.0	22.7	4444.5	2552.9	9001.0	562.6
NHLBI ALL	0 (0/12988)	0 (0/12870)	0.0110 (137/12486)	0 (0/12770)	0 (0/12862)	0.0000770 (1/12994)	0.00177 (23/12994)
EA	0 (0/8698)	0 (0/8530)	0.0111 (93/8354)	0 (0/8460)	0 (0/8506)	0.000116 (1/8600)	0.000116 (21/8600)
AA	0 (0/4390)	0 (0/4340)	0.0104 (44/4232)	0 (0/4310)	0 (0/4356)	0 (0/4394)	0.000455 (2/4394)
1000 Genome ALL	0.000599 (3/5008)	0.0118 (59/5008)	0.126 (633/5008)	0.000799 (4/5008)	0.00140 (7/5008)	0.000200 (1/5008)	0.00160 (9/5008)
AFR	0 (0/1322)	0 (0/1322)	0.0204 (27/1322)	0 (0/1322)	0 (0/1322)	0 (0/1322)	0 (0/1322)
AMR	0 (0/694)	0 (0/694)	0.0115 (8/694)	0 (0/694)	0 (0/694)	0 (0/694)	0.00144 (1/694)
EAS	0.00298 (3/1008)	0.0585 (59/1008)	0.289 (291/1008)	0 (0/1008)	0 (0/1008)	0 (0/1008)	0 (0/1008)
EUR	0 (0/1006)	0 (0/1006)	0.00895 (9/1006)	0 (0/1006)	0.000994 (1/1006)	0 (0/1006)	0.00199 (2/1006)
SAS	0 (0/978)	0 (0/978)	0.305 (298/978)	0.00409 (4/978)	0.00613 (6/978)	0.00102 (1/978)	0.00511 (5/978)
ACB	0 (0/192)	0 (0/192)	0.0105 (2/192)	0 (0/192)	0 (0/192)	0 (0/192)	0 (0/192)
ASW	0 (0/122)	0 (0/122)	0.0328 (4/122)	0 (0/122)	0 (0/122)	0 (0/122)	0 (0/122)
BEB	0 (0/172)	0 (0/172)	0.343 (59/172)	0.00581 (1/172)	0.0174 (3/172)	0.00581 (1/172)	0 (0/172)
CDX	0 (0/186)	0.0269 (5/186)	0.328 (61/186)	0 (0/186)	0 (0/186)	0 (0/186)	0 (0/186)
CEU	0 (0/198)	0 (0/198)	0.0101 (2/198)	0 (0/198)	0.00505 (1/198)	0 (0/198)	0 (0/198)
CHB	0 (0/206)	0.0777 (16/206)	0.286 (59/296)	0 (0/206)	0 (0/206)	0 (0/206)	0 (0/206)
CHS	0 (0/210)	0.0714 (15/210)	0.290 (61/210)	0 (0/210)	0 (0/210)	0 (0/210)	0 (0/210)
CLM	0 (0/188)	0 (0/188)	0 (0/188)	0 (0/188)	0 (0/188)	0 (0/188)	0.00532 (1/188)
ESN	0 (0/198)	0 (0/198)	0.0160 (3/198)	0 (0/198)	0 (0/198)	0 (0/198)	0 (0/198)
FIN	0 (0/198)	0 (0/198)	0 (0/198)	0 (0/198)	0 (0/198)	0 (0/198)	0 (0/198)
GBR	0 (0/182)	0 (0/182)	0.0275 (5/182)	0 (0/182)	0 (0/182)	0 (0/182)	0 (0/182)
GIH	0 (0/206)	0 (0/206)	0.427 (88/206)	0 (0/206)	0 (0/206)	0 (0/206)	0 (0/206)
GWD	0 (0/226)	0 (0/226)	0.00443 (1/226)	0 (0/226)	0 (0/226)	0 (0/226)	0 (0/226)
IBS	0 (0/214)	0 (0/214)	0 (0/214)	0 (0/214)	0 (0/214)	0 (0/214)	0 (0/214)
ITU	0 (0/204)	0 (0/204)	0.260 (53/204)	0.00490 (1/204)	0.0147 (3/204)	0 (0/204)	0 (0/204)
JPT	0.014 (3/208)	0.0577 (12/208)	0.216 (45/208)	0 (0/208)	0 (0/208)	0 (0/208)	0 (0/208)
KHV	0 (0/198)	0.0556 (11/198)	0.328 (65/198)	0 (0/198)	0 (0/198)	0 (0/198)	0 (0/198)
LWK	0 (0/198)	0 (0/198)	0.0707 (14/198)	0 (0/198)	0 (0/198)	0 (0/198)	0 (0/198)
MSL	0 (0/170)	0 (0/170)	0.00588 (1/170)	0 (0/170)	0 (0/170)	0 (0/170)	0 (0/170)
MXL	0 (0/128)	0 (0/128)	0.0156 (2/128)	0 (0/128)	0 (0/128)	0 (0/128)	0 (0/128)
PEL	0 (0/170)	0 (0/170)	0.0118 (2/170)	0 (0/170)	0 (0/170)	0 (0/170)	0 (0/170)
PJL	0 (0/192)	0 (0/192)	0.260 (50/192)	0 (0/192)	0 (0/192)	0 (0/192)	0.0260 (5/192)
PUR	0 (0/208)	0 (0/208)	0.0192 (4/208)	0 (0/208)	0 (0/208)	0 (0/208)	0 (0/208)
STU	0 (0/204)	0 (0/204)	0.235 (48/204)	0.00980 (2/102)	0 (0/204)	0 (0/204)	0 (0/204)
TSI	0 (0/214)	0 (0/214)	0.00935 (2/214)	0 (0/214)	0 (0/214)	0 (0/214)	0.00935 (2/214)
YRI	0 (0/216)	0 (0/216)	0.00926 (2/216)	0 (0/216)	0 (0/216)	0 (0/216)	0 (0/216)

Variant name	p.Ser339Phe	p.Pro369Ser	p.Arg408Gln	p.Gln474Lys	p.Ser503Cys	p.Ala511Val	p.Ile591Thr
dbSNP	rs104895157	rs11466023	rs11466024	rs104895104	rs190705322	rs144270019	rs11466045
ALL	0.000198 (3/18001)	0.00967 (174/18002)	0.00889 (160/18002)	0.000111 (2/18002)	0.000198 (3/18002)	0.000198 (3/18002)	0.00900 (162/18002)
1 in _	6000.3	103.5	112.5	9001.0	6000.7	6000.3	111.1
NHLBI ALL	0.000153 (2/12993)	0.00562 (73/12994)	0.00569 (74/12994)	0.0000770 (1/12994)	0 (0/12994)	0.000231 (3/12994)	0.0108 (140/12994)
EA	0.000233 (2/8600)	0.00674 (58/8600)	0.00674 (58/8600)	0.000116 (1/8600)	0 (0/8600)	0.000349 (3/8600)	0.0152 (131/8600)
AA	0 (0/4393)	0.00341 (15/4394)	0.00364 (16/4394)	0 (0/4394)	0 (0/4394)	0 (0/4394)	0.00205 (9/4394)
1000 Genome ALL	0.000200 (1/5008)	0.0202 (101/5008)	0.0172 (86/5008)	0.000200 (1/5008)	0.000599 (3/5008)	0 (0/5008)	0.00439 (22/5008)
AFR	0 (0/1322)	0.00227 (3/1322)	0.00150 (2/1322)	0 (0/1322)	0 (0/1322)	0 (0/1322)	0 (0/1322)
AMR	0 (0/694)	0.00721 (5/694)	0.00721 (5/694)	0 (0/694)	0 (0/694)	0 (0/694)	0.00432 (3/694)
EAS	0 (0/1008)	0.0675 (68/1008)	0.0546 (55/1008)	0 (0/1008)	0.00298 (3/1008)	0 (0/1008)	0 (0/1008)
EUR	0.000994 (1/1006)	0.00398 (4/1006)	0.00398 (4/1006)	0 (0/1006)	0 (0/1006)	0 (0/1006)	0.0179 (18/1006)
SAS	0 (0/978)	0.0215 (21/978)	0.0205 (20/978)	0.00102 (1/978)	0 (0/978)	0 (0/978)	0.00102 (1/978)
ACB	0 (0/192)	0 (0/192)	0 (0/192)	0 (0/192)	0 (0/192)	0 (0/192)	0 (0/192)
ASW	0 (0/122)	0.00820 (1/122)	0.00820 (1/122)	0 (0/122)	0 (0/122)	0 (0/122)	0 (0/122)
BEB	0 (0/172)	0.0291 (5/172)	0.0291 (5/172)	0 (0/172)	0 (0/172)	0 (0/172)	0.00581 (1/172)
CDX	0 (0/186)	0.0860 (16/186)	0.0806 (15/186)	0 (0/186)	0 (0/186)	0 (0/186)	0 (0/186)
CEU	0 (0/198)	0 (0/198)	0 (0/198)	0 (0/198)	0 (0/198)	0 (0/198)	0.0152 (3/198)
CHB	0 (0/206)	0.0583 (12/206)	0.0388 (8/206)	0 (0/206)	0 (0/206)	0 (0/206)	0 (0/206)
CHS	0 (0/210)	0.0571 (12/210)	0.0476 (10/210)	0 (0/210)	0 (0/210)	0 (0/210)	0 (0/210)
CLM	0 (0/188)	0.00532 (1/188)	0.00532 (1/188)	0 (0/188)	0 (0/188)	0 (0/188)	0.0106 (2/188)
ESN	0 (0/198)	0 (0/198)	0 (0/198)	0 (0/198)	0 (0/198)	0 (0/198)	0 (0/198)
FIN	0 (0/198)	0.0151 (3/198)	0.0160 (3/198)	0 (0/198)	0 (0/198)	0 (0/198)	0.0202 (4/198)
GBR	0 (0/182)	0 (0/182)	0 (0/182)	0 (0/182)	0 (0/182)	0 (0/182)	0.0220 (4/182)
GIH	0 (0/206)	0.00485 (1/206)	0.00485 (1/206)	0 (0/206)	0 (0/206)	0 (0/206)	0 (0/206)
GWD	0 (0/226)	0 (0/226)	0 (0/226)	0 (0/226)	0 (0/226)	0 (0/226)	0 (0/226)
IBS	0 (0/214)	0.00467 (1/214)	0.00467 (1/214)	0 (0/214)	0 (0/214)	0 (0/214)	0.00935 (2/214)
ITU	0 (0/204)	0.0441 (9/204)	0.0392 (8/204)	0 (0/204)	0 (0/204)	0 (0/204)	0 (0/204)
JPT	0 (0/208)	0.0577 (12/208)	0.0529 (11/208)	0 (0/208)	0.0144 (3/208)	0 (0/208)	0 (0/208)
KHV	0 (0/198)	0.0808 (16/198)	0.0556 (11/198)	0 (0/198)	0 (0/198)	0 (0/198)	0 (0/198)
LWK	0 (0/198)	0.00505 (1/197)	0 (0/198)	0 (0/198)	0 (0/198)	0 (0/198)	0 (0/198)
MSL	0 (0/170)	0 (0/170)	0 (0/170)	0 (0/170)	0 (0/170)	0 (0/170)	0 (0/170)
MXL	0 (0/128)	0.00781 (1/128)	0.00781 (1/128)	0 (0/128)	0 (0/128)	0 (0/128)	0.00781 (1/128)
PEL	0 (0/170)	0.0118 (2/170)	0.0118 (2/170)	0 (0/170)	0 (0/170)	0 (0/170)	0 (0/170)
PJL	0 (0/192)	0.0104 (2/192)	0.0104 (2/192)	0 (0/192)	0 (0/192)	0 (0/192)	0 (0/192)
PUR	0 (0/208)	0.00481 (1/208)	0.00481 (1/208)	0 (0/208)	0 (0/208)	0 (0/208)	0 (0/208)
STU	0 (0/204)	0.0196 (4/204)	0.0196 (4/204)	0.00490 (1/204)	0 (0/204)	0 (0/204)	0 (0/204)
TSI	0.00467 (1/214)	0 (0/214)	0 (0/214)	0 (0/214)	0 (0/214)	0 (0/214)	0.0234 (5/214)
YRI	0 (0/216)	0.00463 (1/216)	0.00463 (1/216)	0 (0/216)	0 (0/216)	0 (0/216)	0 (0/216)

Variant name	p.Glu632Ser	p.Arg653His	p.Met680Ile	p.Met694Val	p.Lys695Arg	p.Val722Met	p.Val726Ala	p.Ala744Ser
dbSNP	rs104895128	rs104895085	rs28940580	rs61752717	rs104895094	rs104895201	rs28940579	rs61732874
ALL	0.0000555 (1/18002)	0.0000555 (1/18002)	0.0000555 (1/18002)	0.000222 (4/18001)	0.00233 (42/18002)	0.0000555 (1/18002)	0.00139 (25/18002)	0.00144 (26/18002)
1 in _	18002.0	18002.0	18002.0	4500.3	428.6	18002.0	720.1	692.3
NHLBI ALL	0.0000770 (1/12994)	0 (0/12994)	0.0000770 (1/12994)	0.000231 (3/12994)	0.00254 (33/12994)	0.0000770 (1/12994)	0.00185 (24/12994)	0.00131 (17/12994)
EA	0.000116 (1/8600)	0 (0/8600)	0.000116 (1/8600)	0.000349 (3/8600)	0.00384 (33/8600)	0.000116 (1/8600)	0.00267 (23/8600)	0.00186 (16/8600)
AA	0 (0/4394)	0 (0/4394)	0 (0/4394)	0 (0/4394)	0 (0/4394)	0 (0/4394)	0.000228 (1/4394)	0.000228 (1/4394)
1000 Genome ALL	0 (0/5008)	0.000200 (1/5008)	0 (0/5008)	0.000200 (1/5008)	0.00180 (9/5008)	0 (0/5008)	0.000200 (1/5008)	0.00180 (9/5008)
AFR	0 (0/1322)	0 (0/1322)	0 (0/1322)	0 (0/1322)	0 (0/1322)	0 (0/1322)	0 (0/1322)	0 (0/1322)
AMR	0 (0/694)	0 (0/694)	0 (0/694)	0.00144 (1/694)	0.00288 (2/694)	0 (0/694)	0 (0/694)	0.00576 (4/694)
EAS	0 (0/1008)	0 (0/1008)	0 (0/1008)	0 (0/1008)	0 (0/1008)	0 (0/1008)	0 (0/1008)	0 (0/1008)
EUR	0 (0/1006)	0.000994 (1/1006)	0 (0/1006)	0 (0/1006)	0.00696 (7/1006)	0 (0/1006)	0.000994 (1/1006)	0.00497 (5/1006)
SAS	0 (0/978)	0 (0/978)	0 (0/978)	0 (0/978)	0 (0/978)	0 (0/978)	0 (0/978)	0 (0/978)
ACB	0 (0/192)	0 (0/192)	0 (0/192)	0 (0/192)	0 (0/192)	0 (0/192)	0 (0/192)	0 (0/192)
ASW	0 (0/122)	0 (0/122)	0 (0/122)	0 (0/122)	0 (0/122)	0 (0/122)	0 (0/122)	0 (0/122)
BEB	0 (0/172)	0 (0/172)	0 (0/172)	0 (0/172)	0 (0/172)	0 (0/172)	0 (0/172)	0 (0/172)
CDX	0 (0/186)	0 (0/186)	0 (0/186)	0 (0/186)	0 (0/186)	0 (0/186)	0 (0/186)	0 (0/186)
CEU	0 (0/198)	0 (0/198)	0 (0/198)	0 (0/198)	0 (0/198)	0 (0/198)	0 (0/198)	0 (0/198)
CHB	0 (0/206)	0 (0/206)	0 (0/206)	0 (0/206)	0 (0/206)	0 (0/206)	0 (0/206)	0 (0/206)
CHS	0 (0/210)	0 (0/210)	0 (0/210)	0 (0/210)	0 (0/210)	0 (0/210)	0 (0/210)	0 (0/210)
CLM	0 (0/188)	0 (0/188)	0 (0/188)	0 (0/188)	0 (0/188)	0 (0/188)	0 (0/188)	0.0106 (2/188)
ESN	0 (0/198)	0 (0/198)	0 (0/198)	0 (0/198)	0 (0/198)	0 (0/198)	0 (0/198)	0 (0/198)
FIN	0 (0/198)	0 (0/198)	0 (0/198)	0 (0/198)	0.0253 (5/198)	0 (0/198)	0 (0/198)	0 (0/198)
GBR	0 (0/182)	0 (0/182)	0 (0/182)	0 (0/182)	0 (0/182)	0 (0/182)	0 (0/182)	0 (0/182)
GIH	0 (0/206)	0 (0/206)	0 (0/206)	0 (0/206)	0 (0/206)	0 (0/206)	0 (0/206)	0 (0/206)
GWD	0 (0/226)	0 (0/226)	0 (0/226)	0 (0/226)	0 (0/226)	0 (0/226)	0 (0/226)	0 (0/226)
IBS	0 (0/214)	0.00467 (1/214)	0 (0/214)	0 (0/214)	0.00467 (1/214)	0 (0/214)	0 (0/214)	0.0187 (4/214)
ITU	0 (0/204)	0 (0/204)	0 (0/204)	0 (0/204)	0 (0/204)	0 (0/204)	0 (0/204)	0 (0/204)
JPT	0 (0/208)	0 (0/208)	0 (0/208)	0 (0/208)	0 (0/208)	0 (0/208)	0 (0/208)	0 (0/208)
KHV	0 (0/198)	0 (0/198)	0 (0/198)	0 (0/198)	0 (0/198)	0 (0/198)	0 (0/198)	0 (0/198)
LWK	0 (0/198)	0 (0/198)	0 (0/198)	0 (0/198)	0 (0/198)	0 (0/198)	0 (0/198)	0 (0/198)
MSL	0 (0/170)	0 (0/170)	0 (0/170)	0 (0/170)	0 (0/170)	0 (0/170)	0 (0/170)	0 (0/170)
MXL	0 (0/128)	0 (0/128)	0 (0/128)	0 (0/128)	0 (0/128)	0 (0/128)	0 (0/128)	0 (0/128)
PEL	0 (0/170)	0 (0/170)	0 (0/170)	0.00588 (1/170)	0 (0/170)	0 (0/170)	0 (0/170)	0 (0/170)
PJL	0 (0/192)	0 (0/192)	0 (0/192)	0 (0/192)	0 (0/192)	0 (0/192)	0 (0/192)	0 (0/192)
PUR	0 (0/208)	0 (0/208)	0 (0/208)	0 (0/208)	0.00962 (2/208)	0 (0/208)	0 (0/208)	0.00962 (2/208)
STU	0 (0/204)	0 (0/204)	0 (0/204)	0 (0/204)	0 (0/204)	0 (0/204)	0 (0/204)	0 (0/204)
TSI	0 (0/214)	0 (0/214)	0 (0/214)	0 (0/214)	0.00467 (1/214)	0 (0/214)	0.00467 (1/214)	0.00467 (1/214)
YRI	0 (0/216)	0 (0/216)	0 (0/216)	0 (0/216)	0 (0/216)	0 (0/216)	0 (0/216)	0 (0/216)

### Carrier rate variability by mutation type and ethnicity

Among the 22 *MEFV* mutations detected, the most common was E148Q, with a frequency of 1 in 22.7 (4.40%) (Table2). The allele frequencies of the other four founder mutations were as follows: M680I, 0.00555%; M694I, 0%; M694V, 0.0222%; and V726A, 0.139% (Table2). The prevalence rates are theoretically estimated to be equivalent to the squares of these carrier rates. Previous studies demonstrate that E148Q is not likely to be a disease-causing mutation (Tchernitchko et al. 2003, 2006; Giancane et al. 2015) and the high carrier rate of E148Q in this research (Table2) was consistent with the previous data. Thus, E148Q is excluded from following analysis.

**Figure 2:**
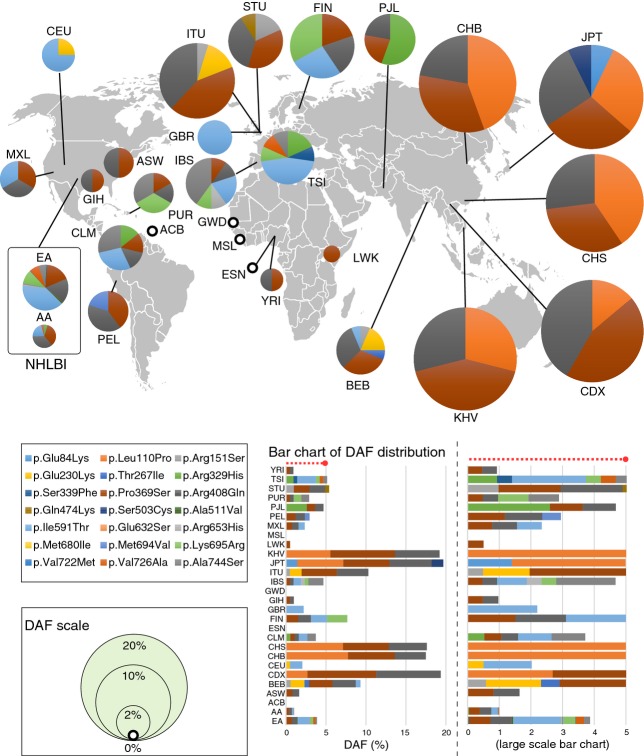
Geographical distribution of derived allele frequencies for *MEFV* mutations of FMF. The pie areas are proportional to derived allele frequency (DAF) of the 21 (except E148Q) *MEFV* mutations of FMF. The 1000G and NHLBI (26 + 2) populations are displayed separately. The bar chart of DAF distribution is described in the lower right panel.

Carrier frequencies for disease-causing variants vary significantly by racial and ethnic groups (Lazarin et al. 2013). Figure2 shows a global map of the DAF distribution of the 21 *MEFV* mutations for FMF except E148Q. Among the other four founder mutations, M680I was Caucasian-specific, and V726A was much more prevalent in Caucasian (Table2). In East Asian populations, the average DAF was 18.7% (CDX, 19.4%; CHB, 17.5%; CHS, 17.6%; JPT, 19.7%) (Fig.2 and Table2). Also, the major variant in Europeans (NHLBI: 1.52%; 1000G: 1.79%) and Hispanics (1000G: 0.432%) was I591T. A recently available database, ExAC, with more than 60,000 exomes was additionally searched for 57 *MEFV* mutations (Table3) although this database does not provide the detailed information about country and ethnic group of individuals. A total of 31 *MEFV* mutations were detected (Table3) and the similar DAF distribution was obtained between ExAC and 1000 Genome + NHLBI data (Table2 and 3) as a whole.

**Table 3:** Estimated carrier rates of FMF using ExAC

Mutation	dbSNP	East Asian	South Asian	Latino	European (Non-Finnish)	European (Finnish)	African	Other
p.Glu84Lys	rs150819742	0.00151 (13/8588)	0.0000609 (1/16418)	0 (0/11480)	0 (0/64378)	0 (0/6316)	0 (0/9498)	0 (0/864)
p.Leu110Pro	rs11466018	0.08465 (717/8470)	0.000793 (13/16386)	0.000349 (4/11458)	0.000159 (10/62936)	0 (0/6602)	0 (0/9144)	0.00115 (1/870)
p.Ser141Ile	rs104895130	0 (0/7806)	0 (0/15912)	0 (0/10018)	0.0000183 (1/54662)	0 (0/5548)	0 (0/7550)	0.00132 (1/756)
p.Glu148Gln	rs3743930	0.315 (2275/7222)	0.302 (4688/15536)	0.0218 (184/8460)	0.0197 (961/48764)	0.00129 (6/4668)	0.0184 (124/6734)	0.0716 (49/684)
p.Glu148Val	rs104895076	0 (0/6392)	0.000474 (7/14762)	0 (0/8234)	0.0000416 (2/48076)	0 (0/4594)	0 (0/6636)	0 (0/668)
p.Arg151Ser	rs104895185	0 (0/5646)	0.000712 (10/14052)	0 (0/7110)	0 (0/43120)	0 (0/3968)	0 (0/5938)	0 (0/612)
p.Ser166Leu		0 (0/1864)	0.000897 (9/10030)	0 (0/1514)	0 (0/14194)	0 (0/930)	0 (0/2048)	0 (0/264)
p.Glu167Asp	rs104895079	0 (0/1704)	0 (0/9872)	0 (0/1378)	0.0000756 (1/13236)	0 (0/842)	0 (0/1896)	0 (0/246)
p.Ser179Asn		0 (0/1368)	0.00179 (17/9516)	0 (0/1070)	0.0000881 (1/11352)	0 (0/644)	0 (0/1552)	0 (0/228)
p.Glu230Lys	rs104895080	0 (0/8570)	0.00431 (71/16486)	0.000260 (3/11540)	0.0000907 (6/66162)	0 (0/6606)	0 (0/10240)	0 (0/898)
p.Thr267Ile	rs104895081	0 (0/8644)	0.000363 (6/16512)	0.000173 (2/11574)	0.000135 (9/66732)	0 (0/6612)	0 (0/10392)	0 (0/906)
p.Arg329His	rs104895112	0 (0/8630)	0.00228 (37/16258)	0.000522 (6/11504)	0.00223 (147/65936)	0 (0/6396)	0.000293 (3/10248)	0.00112 (1/892)
p.Ser339Phe	rs104895157	0 (0/8610)	0.000185 (3/16246)	0 (0/11474)	0.000274 (18/65636)	0 (0/6394)	0.000197 (2/10148)	0 (0/890)
p.Arg354Trp	rs104895116	0 (0/8562)	0.0000613 (1/16300)	0 (0/11482)	0.0000611 (4/65466)	0 (0/6434)	0 (0/10158)	0 (0/880)
p.Pro369Ser	rs11466023	0.0716 (616/8608)	0.0147 (241/16442)	0.00468 (54/11536)	0.00975 (645/66174)	0.0155 (101/6522)	0.00397 (41/10318)	0.0189 (17/902)
p.Arg408Gln	rs11466024	0.0541 (465/8600)	0.0144 (237/16428)	0.00434 (50/11518)	0.00973 (639/65696)	0.0153 (101/6596)	0.00419 (42/10016)	0.0190 (17/896)
p.Gln474Lys	rs104895104	0 (0/8652)	0.0000606 (1/16512)	0 (0/11578)	0 (0/66724)	0 (0/6614)	0 (0/10404)	0 (0/908)
p.Phe479Leu	rs104895083	0 (0/8654)	0 (0/16512)	0 (0/11578)	0.0000599 (4/66740)	0 (0/6614)	0 (0/10406)	0 (0/908)
p.Ser503Cys	rs190705322	0.00162 (14/8654)	0 (0/16512)	0 (0/11578)	0 (0/66738)	0 (0/6614)	0 (0/10402)	0 (0/908)
p.Ala511Val	rs144270019	0 (0/8654)	0 (0/16512)	0 (0/11578)	0.0000749 (5/66718)	0 (0/6614)	0 (0/10400)	0 (0/908)
p.Ile591Thr	rs11466045	0.000116 (1/8594)	0.00335 (55/16402)	0.00290 (33/11374)	0.0147 (976/66284)	0.0215 (141/6564)	0.00176 (18/10254)	0.0100 (9/898)
p.Glu632Ser	rs104895128	0 (0/8636)	0 (0/14474)	0 (0/11556)	0.0000456 (3/65754)	0 (0/6614)	0 (0/10308)	0 (0/862)
p.Arg653His	rs104895085	0 (0/8654)	0.0000608 (1/16444)	0 (0/11576)	0.0000300 (2/66624)	0 (0/6614)	0.000289 (3/10386)	0 (0/906)
p.Gly678Glu	rs104895088	0 (0/8654)	0 (0/16512)	0 (0/11576)	0.0000450 (3/66740)	0 (0/6614)	0 (0/10406)	0 (0/908)
p.Met680Ile (G→C)	rs28940580	0 (0/8654)	0 (0/16512)	0 (0/11576)	0.000150 (10/66740)	0 (0/6614)	0 (0/10406)	0.00110 (1/908)
p.Met680Ile (G→A)	rs28940580	0 (0/8654)	0 (0/16512)	0 (0/11576)	0.0000300 (2/66740)	0 (0/6614)	0 (0/10406)	0 (0/908)
p.Met694Val	rs61752717	0 (0/8654)	0 (0/16512)	0.000432 (5/11576)	0.000285 (19/66738)	0 (0/6614)	0.0000961 (1/10406)	0.00330 (3/908)
p.Lys695Arg	rs104895094	0 (0/8654)	0.0000606 (1/16512)	0.00225 (26/11576)	0.00791 (528/66740)	0.0161 (107/6614)	0.0000961 (1/10406)	0.00551 (5/908)
p.Val722Met	rs104895201	0 (0/8654)	0 (0/16512)	0 (0/11578)	0.0000450 (3/66736)	0 (0/6614)	0 (0/10406)	0 (0/908)
p.Val726Ala	rs28940579	0 (0/8654)	0.000121 (2/16512)	0.000173 (2/11578)	0.0000450 (217/66736)	0 (0/6614)	0 (0/10406)	0.00330 (3/908)
p.Ala744Ser	rs61732874	0 (0/8654)	0.000363 (6/16512)	0.00181 (21/11578)	0.00214 (143/66728)	0.000756 (5/6614)	0.000385 (4/10404)	0.00110 (1/906)
Total (All/except E148Q)		0.529/0.214	0.347/0.0450	0.0397/0.0179	0.0679/0.0482	0.0704/0.0692	0.0297/0.0123	0.137/0.0658

### Consistency of data between two different exomes

To determine the validity of this methodology, I examined the extent of differences in the two exome-based prevalence rates by comparing DAFs in African and European ancestries between the 1000G and NHLBI datasets (Table S1). A pairwise proportions test was used to test the null hypothesis that the proportions in the two estimates were identical. This formula is referred to as a *Z* test because the statistic is as follows:

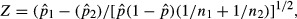


where 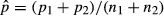 and the indices (1, 2) refer to the first and second lines of the table. A pairwise proportion test between the two exome resources showed no significant differences between the two different exomes (43 cases; *P *≫ 0.05), except in one case (*P *<* *0.05) (Table S1). This finding suggests that exome-based predictions are free of most confounding factors (such as diagnostic criteria, instrument, skill, and screening rate) and may be a more objective indicator.

## Discussion

Although genetic research into FMF began in 1990s (The French International FMF consortium 1997; The International FMF Consortium 1997), we still lack a complete picture of its genetic variation, carrier frequency, and penetrance. Exome-based epidemiology is a promising alternative method for genetic epidemiology because it provides information on both common and rare mutations in large numbers of individuals. Using exome data from a total of 9007 individuals from the two largest population exomes, I established a reliable epidemiology of FMF mutations with a small margin of error.

### Estimated prevalence rates are considerably higher than those seen in clinical practice

There are only little data of carrier frequencies for comparison with this study because previous studies analyzed individuals mainly from some Mediterranean countries. Hofer et al. (2006) reported FMF prevalence rates in individuals from Western countries on a mass scale. The relative frequencies of the mutations found in this study [NHLBI EA, 4.98% (3.87%; except E148Q); 1000G EUR, 5.27% (4.38%; except E148Q) (Table2)] are considerably higher than the results of Hofer et al. (2006) who reported a prevalence of as 2.5 per 100,000 (0.004%) people even if E148Q is excluded in this research. In another study in Japanese populations, carrier frequencies [1000G JPT, 41.3% (19.7%; except E148Q) (Table2)] were much higher than the clinical incidence rate (0.000417%; http://www.nanbyou.or.jp/entry/3238). The variant T267I was frequently detected in Bangladesh (0.581% Table2), where FMF patients were not reported. These results suggest that some *MEFV* mutations, including E84K, L110P, E148Q, T267I, P369S, R408Q, S503C, and I591T, are polymorphisms, not disease-causing mutations. It has been proposed that E148Q is likely to be a polymorphism, not a disease-causing mutation, and has low penetrance (Tchernitchko et al. 2003, 2006) and my findings agree with these research results. Furthermore, the result here suggested the nonpenetrance of some other mutations. There is often the case where the causative mutations are determined too easily without analyzing potential effect of mutations (Cooper et al. 2013; van Rheenen et al. 2014; Siemiatkowska et al. 2014). Discordance between DAF and incidence rate may be caused by another infrequent mutation closely linked to these mutations. Another promising hypothesis is that FMF is a multifactorial and polygenic genetic disorder associated with the effects of multiple genes in combination with lifestyle and environmental factors.

In conclusion, exome-based epidemiology revealed the country-by-country carrier rates of FMF with respect to each mutation and provided a clue to understand the penetrance and screening priority of each mutation. An unexpectedly high carrier rate of FMF in Europeans and Asians raises the strong possibility that some *MEFV* mutations may be benign variants with few or no pathological significance. This study highlights the need for caution in interpreting genetic tests in FMF patients. Similar method could be used to uncover the incomplete or no penetrance of mutations in genetic disorders.

## Supporting Information

**Table S1.** Comparison of epidemiological data between two different exomes (1000G and NHLBI).
