# Supplementary material for: Global epidemiology of Familial Mediterranean fever mutations using population exome sequences
Source: Mol Genet Genomic Med. 2015 Apr 5;3(4):272–82. doi: 10.1002/mgg3.140 (PMC4521964; doi:10.1002/mgg3.140)
Supplement: Supplementary file 1 [file mgg30003-0272-sd1.doc]

Supporting Information

**Global epidemiology of Familial Mediterranean fever using population exome sequences**

**Kohei Fujikura^1^***

^1^ Kobe University Hospital, 7-5-1, Kusunoki-cho, Chuo-ku, Kobe 650-0017, Japan

*****Correspondence should be addressed to Kohei Fujikura, Kobe University Hospital, 7-5-1, Kusunoki-cho, Chuo-ku, Kobe 650-0017, Japan.

Telephone: +81-90-3906-9772

Fax

Email: [kofujikura@gmail.com](mailto:kofujikura@gmail.com)

**Table S1.** **Comparison of epidemiological data between two different exomes (1000G and NHLBI)**

**
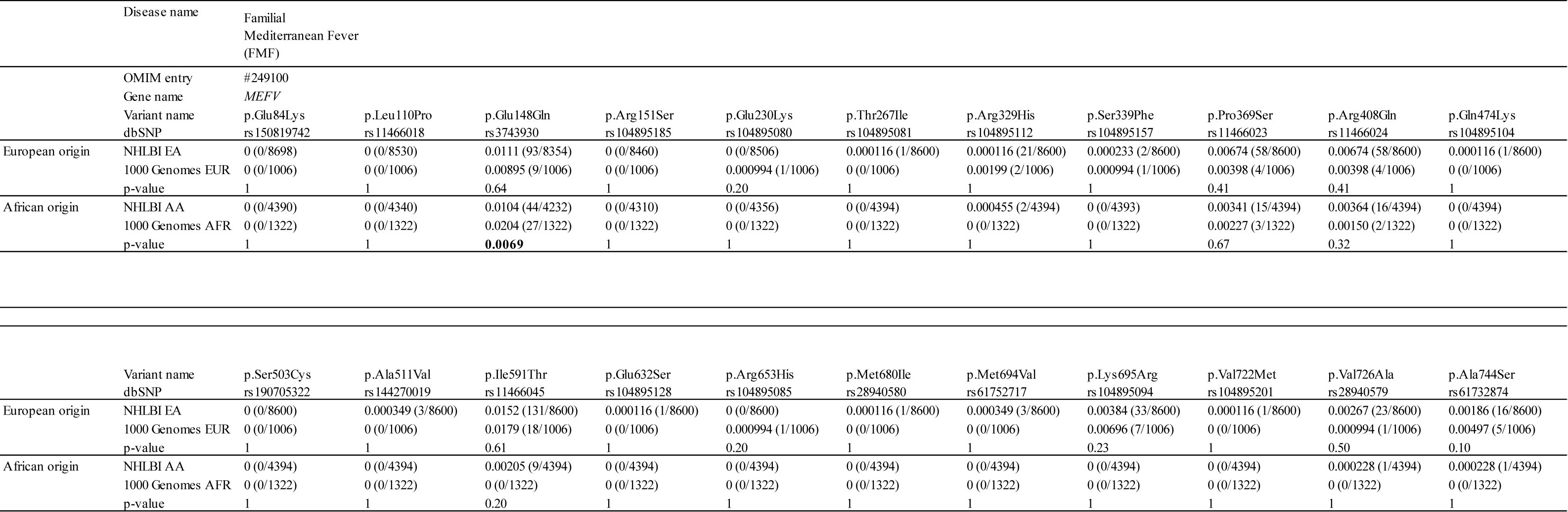
**

The *P*-value is calculated from pairwise proportion tests of allele frequencies in European and African ancestries between two different exome resources (1000G and NHLBI).

**Supplementary references**

1. Bernot A, da Silva C, Petit JL, et al. Non-founder mutations in the MEFV gene establish this gene as the cause of familial Mediterranean fever (FMF). Human molecular genetics 1998;7:1317-25.

2. Booth DR, Gillmore JD, Booth SE, Pepys MB, Hawkins PN. Pyrin/marenostrin mutations in familial Mediterranean fever. QJM : monthly journal of the Association of Physicians 1998;91:603-6.

3. Cazeneuve C, Sarkisian T, Pecheux C, et al. MEFV-Gene analysis in armenian patients with Familial Mediterranean fever: diagnostic value and unfavorable renal prognosis of the M694V homozygous genotype-genetic and therapeutic implications. American journal of human genetics 1999;65:88-97.

4. Dode C, Pecheux C, Cazeneuve C, et al. Mutations in the MEFV gene in a large series of patients with a clinical diagnosis of familial Mediterranean fever. American journal of medical genetics 2000;92:241-6.

5. Dode C, Pecheux C, Cazeneuve C, et al. Mutations in the MEFV gene in a large series of patients with a clinical diagnosis of familial Mediterranean fever. American journal of medical genetics 2000;92:241-6.

6. Notarnicola C, Manna R, Rey JM, Touitou I. Y688X, the first nonsense mutation in familial Mediterranean fever (FMF). Human mutation 2001;17:79.

7. Timmann C, Muntau B, Kuhne K, Gelhaus A, Horstmann RD. Two novel mutations R653H and E230K in the Mediterranean fever gene associated with disease. Mutation research 2001;479:235-9.

8. Tchernitchko D, Legendre M, Delahaye A, et al. Clinical evaluation of a reverse hybridization assay for the molecular detection of twelve MEFV gene mutations. Clinical chemistry 2003;49:1942-5.

9. Timmann C, Mersinli O, Kuhne K, et al. Familial Mediterranean fever with amyloidosis associated with novel exon 2 mutation (S1791) of the MEFV gene. Blood cells, molecules & diseases 2003;31:320-3.

10. Cazeneuve C, Papin S, Jeru I, Duquesnoy P, Amselem S. Subcellular localisation of marenostrin/pyrin isoforms carrying the most common mutations involved in familial Mediterranean fever in the presence or absence of its binding partner ASC. Journal of medical genetics 2004;41:e24.

11. Medlej-Hashim M, Serre JL, Corbani S, et al. Familial Mediterranean fever (FMF) in Lebanon and Jordan: a population genetics study and report of three novel mutations. European journal of medical genetics 2005;48:412-20.

12. Medlej-Hashim M, Serre JL, Corbani S, et al. Familial Mediterranean fever (FMF) in Lebanon and Jordan: a population genetics study and report of three novel mutations. European journal of medical genetics 2005;48:412-20.

13. Goulielmos GN, Fragouli E, Aksentijevich I, Sidiropoulos P, Boumpas DT, Eliopoulos E. Mutational analysis of the PRYSPRY domain of pyrin and implications for familial mediterranean fever (FMF). Biochemical and biophysical research communications 2006;345:1326-32.

14. Tomiyama N, Higashiuesato Y, Oda T, et al. MEFV mutation analysis of familial Mediterranean fever in Japan. Clinical and experimental rheumatology 2008;26:13-7.

15. Aksentijevich I, Kastner DL. Genetics of monogenic autoinflammatory diseases: past successes, future challenges. Nature reviews Rheumatology 2011;7:469-78.

16. Berdeli A, Mir S, Nalbantoglu S, et al. Comprehensive analysis of a large-scale screen for MEFV gene mutations: do they truly provide a "heterozygote advantage" in Turkey? Genetic testing and molecular biomarkers 2011;15:475-82.

17. Cornelius N, Duno M. Molecular evaluation of 458 patients referred with a clinical diagnosis of familial Mediterranean fever in Scandinavia. Rheumatology international 2011;31:1531-3.

18. Shohat M, Halpern GJ. Familial Mediterranean fever--a review. Genetics in medicine : official journal of the American College of Medical Genetics 2011;13:487-98.

19. Federici S, Calcagno G, Finetti M, et al. Clinical impact of MEFV mutations in children with periodic fever in a prevalent western European Caucasian population. Annals of the rheumatic diseases 2012;71:1961-5.

20. Kalkan G, Demirkaya E, Acikel CH, et al. Evaluation of the current disease severity scores in paediatric FMF: is it necessary to develop a new one? Rheumatology 2012;51:743-8.

21. Oztuzcu S, Ulasli M, Ergun S, et al. Screening of common and novel familial mediterranean fever mutations in south-east part of Turkey. Molecular biology reports 2014;41:2601-7.
